# Tumor-derived exosomal HMGB1 promotes esophageal squamous cell carcinoma progression through inducing PD1^+^ TAM expansion

**DOI:** 10.1038/s41389-019-0126-2

**Published:** 2019-02-22

**Authors:** Bin Li, Tie-Niu Song, Fu-Rong Wang, Ci Yin, Zheng Li, Jun-Ping Lin, Yu-Qi Meng, Hai-Ming Feng, Tao Jing

**Affiliations:** 1 Department of Thoracic Surgery, Lanzhou University Second Hospital, Lanzhou University Second Clinical Medical College, Lanzhou University, 730030 Lanzhou, China; 2 Gansu Provincial Key Laboratory of Digestive System Tumors, Lanzhou University Second Hospital, Lanzhou University Second Clinical Medical College, Lanzhou University, 730030 Lanzhou, China; 3 Department of Pathology, Lanzhou University Second Hospital, Lanzhou University Second Clinical Medical College, Lanzhou University, 730030 Lanzhou, China

## Abstract

Macrophages constitute one of the most common components of immune cells, which penetrate tumors and they have a key role in tumor prognosis. Here, we identified an unrecognized macrophage subpopulation, which favors tumorigenesis. These macrophages express programmed cell death protein 1 (PD1) in a constitutive manner and accumulates in esophageal squamous cell carcinoma (ESCC) in advanced stage of the disease and is negatively associated with the survival of ESCC patients. The PD1^+^ tumor-associated macrophages (PD1^+^ TAMs) displayed surface pattern and function akin to M2: a substantial enhancement in CD206 and IL-10 expression; a specific reduction in HLA-DR, CD64, and IL-12 expression; and a significant increase in the ability to inhibit CD8^+^ T-cell proliferation. Triggering of PD1 signal is effective in increasing PD1^+^ TAM function. Moreover, exosomal HMGB1 obtained from tumors are efficient in triggering differentiation of monocytes into PD1^+^ TAMs, which display phenotypic and functional properties of M2. Overall, our work is the first finding to confirm that exosomal HMGB1 obtained from ESCC can successfully trigger clonal expansion of PD1^+^ TAM. Further, as the macrophages exhibit an M2-like surface profile and function, thereby creating conditions for development of ESCC. Thus, effective methods of treatment include combining immunotherapy with targeting PD1^+^ TAMs and tumor-derived exosomal HMGB1 to resuscitate immune function in individuals suffering from ESCC.

## Introduction

Esophageal cancer has emerged as the eighth most prevalent type of cancer and also the sixth most common reason of cancer-associated death in the world^1^. Asian population displays esophageal squamous cell carcinoma (ESCC) as the predominant histological form of the disease. Although multimodal treatments including surgery, radiotherapy, and chemotherapy are currently being utilized, ESCC still present inferior prognostic features, usually with a survival rate of 5 years ranging from 10 to 25%^2–4^. A growing number of evidences confirm that the tumor microenvironment contains diverse cell populations that interact with cancer cells and participate in all stages of tumorigenesis^5^. Tumor-infiltrating immune cells and immune responses within the tumor microenvironment are promising therapeutic targets. PD1 is an extensively researched and clinically successful target for immune-regulatory drugs^6^.

Macrophages (Mφs), which are one of most common components of tumor-infiltrating immune cells^7,8^, have a key role in tumor prognosis^9^. Present research highlights involvement of TAMs in tumor angiogenesis^10^ and metastasis^11^, and inhibits T-cell function via secretion of cytokines^12,13^. Phenotypic plasticity is a hallmark of TAMs, which often polarize towards M1 and M2 states^14^. Our previous study, along with other studies, confirmed that macrophages express high levels of PD1 during tumor progression and in the context of pathogenic infection^15–19^, which are targeted by M2 macrophages. However, PD1 is expressed by TAMs in ESCC and mechanism behind induction of PD1^+^ TAMs are still unknown.

Exosomes, the tiny intraluminal vesicles having diameters in the range of 30 to 200 nm, have been documented to bring about intercellular communication at both local and systemic levels. Exosomes trade information in the form of various biomolecules like mRNAs, proteins, microRNAs, and cDNA^20–23^. Immune-regulatory functions of exosomes are increasingly being studied^24–37^. Under such scenario, we hypothesized that exosomes secreted from tumor microenvironment are involved in PD1^+^ TAM expansion during development of ESCC.

## Results

### PD1^+^ TAMs are abundant with ESCC tissues and negatively correlated with the survival of patients

**Fig. 1:**
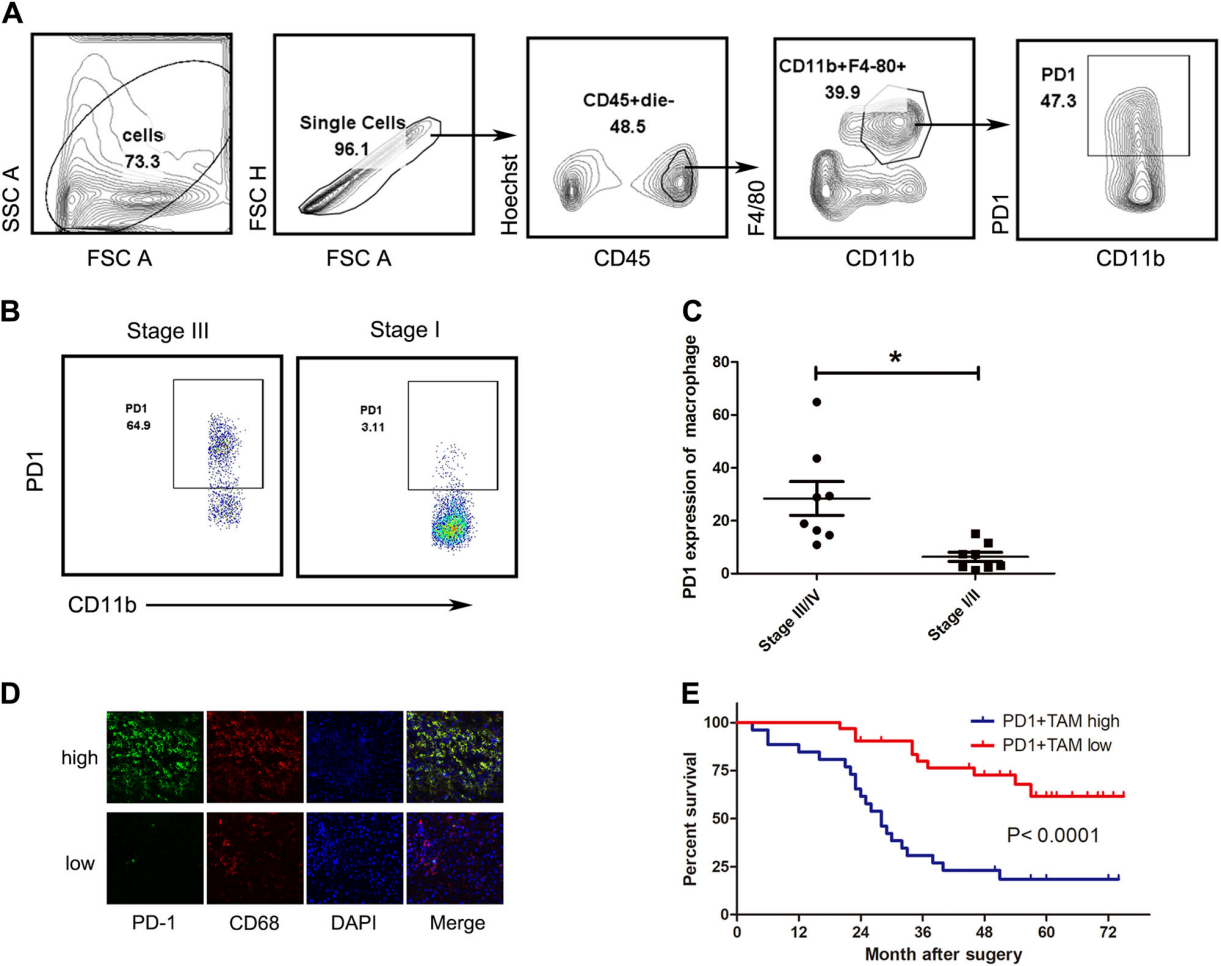
PD1^+^ TAMs are enriched in ESCC tissues and negatively associated with the survival of patients. **a** Gating strategy for flow cytometry to enumerate macrophages infiltrating ESCC. Debris and doublets were excluded, followed by quantification of TAMs as CD45^+^ Hoechst^−^ F4/80^+^ CD11b^+^. **b** PD1 level estimation in TAMs from ESCC tissues. Representative graphs of PD1 levels in TAMs are shown. **c** Associations of PD1^+^ TAMs with ESCC patients’ TNM stage are shown. **d** Immunofluorescence analysis of PD1 (green) and CD68 (red) expression levels in ESCC tissue. **e** Association of tumor PD1^+^ TAMs with patient survival. *p < 0.01, **p < 0.001 (Student’s *t*-test)

We analyzed the PD1 levels in TAMs in 16 ESCC tissues by flow cytometry. The results showed that TAMs expressed PD1 (Fig. 1a, b) and the frequency of PD1^+^ TAMs was positively associated with disease progression in patients with ESCC (Fig. 1c). Furthermore, immunofluorescence of ESCC tissues confirmed that TAMs expressed PD1 (Fig. 1d). According to the median value of PD1 density in macrophages, ESCC patients who underwent curative resection with follow-up data were divided into PD1^+^ TAM high group (*n* = 26, Table 1) and PD1^ +^ TAM low group (*n* = 31, Table 1), and the readouts observed negative association between the frequency of PD1^+^ TAMs and the survival of patients with ESCC (Fig. 1e).

**Table 1:** Correlation between PD1^+^ TAM and various clinicopathological parameters in ESCC patients

	Low (*n* = 31)	High (*n* = 26)
Age, years, median (range)	59 (38–73)	62 (37–75)
Gender, *n* (%)		
Male	23 (74)	22 (85)
Female	8 (26)	4 (15)
pT, *n* (%)		
1	15 (48.4)	5 (19.2)
2	12 (38.7)	11 (42.3)
3	4 (12.9)	10 (38.5)
pN, *n* (%)		
1	17 (54.8)	5 (19.2)
2	12 (38.7)	13 (50.0)
3	3 (6.5)	8 (30.8)
pStage, *n* (%)		
I/II	23 (74)	8 (30.7)
III/IV	8 (26)	18 (69.3)

### PD1^+^ TAMs in patients with ESCC exhibit M2 macrophage characteristics

**Fig. 2:**
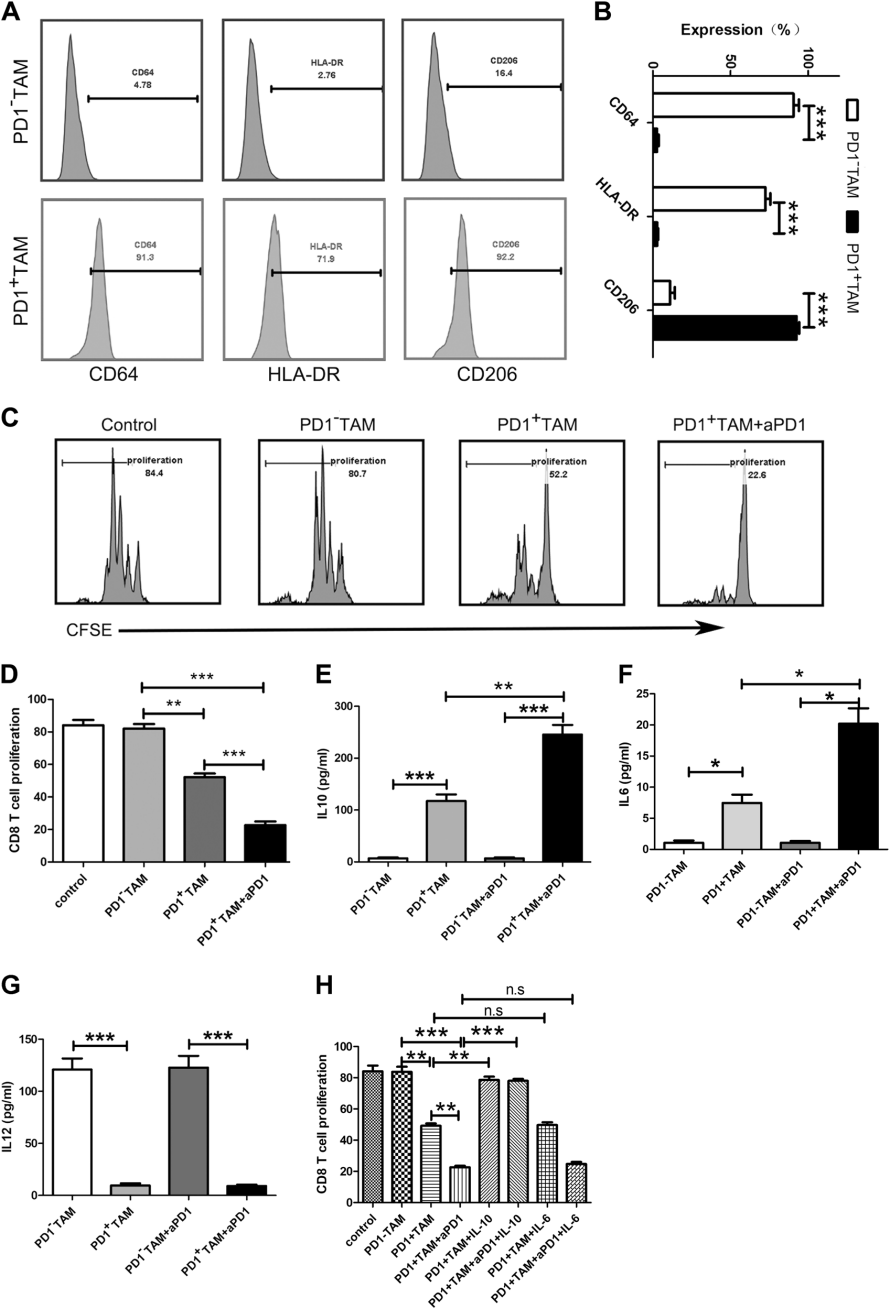
PD1^+^ TAMs in patients with ESCC exhibit an M2 macrophage characteristics. **a**, **b** Flow cytometry helped to determine the levels of CD64, HLA-DR, and CD206 in PD1^−^ and PD1^+^ TAMs from ESCC tissues. Flow cytometry graphs are shown in **a**. Evaluation of hallmark molecules expressed by PD1^−^ and PD1^+^ TAMs. **b** Data represent the average of the three experiments performed independently. **c**, **d** CD8^+^ T-cell proliferation in control (CD8^+^ T cells were cultured alone), PD1^−^ TAM (CD8^+^ T cells were co-cultured with PD1^-^ TAMs), PD1^+^ TAM (CD8^+^ T cells were co-cultured with PD1^+^ TAMs), and PD1^+^ TAM + aPD1 (CD8^+^ T cells were co-cultured with PD1^+^ TAMs that were treated with aPD1) was evaluated. Representative graphs are shown in **c**. **d** Data represent the average of the three experiments performed independently. **e**–**g** Analysis of IL-10, IL-6, and IL-12 levels in PD1^−^ and PD1^+^ TAMs obtained from ESCC tissues by ELISA. **h** The multiplication of CD8^+^ T cells in control (CD8^+^ T cells were cultured alone), PD1^−^ TAM (CD8^+^ T cells were co-cultured with PD1^−^ TAMs), PD1^+^ TAM (CD8^+^ T cells were co-cultured with PD1^+^ TAMs), PD1^+^ TAM + aIL10 (CD8^+^ T cells were co-cultured with PD1^+^ TAMs and anti-IL-10 Ab was added), PD1^+^ TAM + aIL6 (CD8^+^ T cells were co-cultured with PD1^+^ TAMs and anti-IL-6 Ab was added), PD1^+^ TAM + aPD1 (CD8^+^ T cells were co-cultured with PD1^+^ TAMs that were treated with aPD1), PD1^+^ TAM + aPD1 + aIL10 (CD8^+^ T cells were co-cultured with PD1^+^ TAMs that were treated with aPD1 anti-IL-10 Ab was added) and PD1^+^ TAM + aPD1 + aIL6 (CD8^+^ T cells were co-cultured with PD1^+^ TAMs that were treated with aPD1 anti-IL-6 Ab was added) was analyzed. Data represent the average of three independent experiments. *p < 0.05, **p < 0.01, ***p < 0.001 (Student’s *t*-test)

To describe the features of PD1^+^ TAMs, our team investigated their surface molecules, patterns of cytokine expression, as well as biological function by multiple complementary strategies. We first analyzed the typical M1 and M2 markers of TAMs by flow cytometry, and the results showed that PD1^+^ TAMs expressed more CD206, and less CD64 and HLA-DR than PD1^−^ TAMs (Fig. 2a, b). Moreover, we determined the cytokine expression patterns of TAMs through ELISA, and the results confirmed the PD1^+^ TAMs produced elevated levels of M2 marker IL-10 (Fig. 2e), whereas M1 marker IL-12 was not found to be high (Fig. 2f). Next, we determined the biological function of TAMs using MLR, and the results displayed that PD1^+^ TAMs remarkably inhibited the expansion of CD8^+^ T cells (Fig. 2c, d). In addition, we investigated whether the PD1 signal contributed to the cytokine expression patterns and biological function of PD1^+^ TAMs, and the results showed that a PD1-specific agonist significantly promoted the IL-10 and IL-6 expression of PD1^+^ TAMs (Fig. 2e, f), but did not influence IL-12 expression (Fig. 2g). Moreover, it enhanced the ability of PD1^+^ TAMs to impair CD8^+^ T-cell proliferation (Fig. 2c, d). Anti-IL-10-blocking Ab promoted CD8^+^ T-cell proliferation, while anti-IL-6-blocking Ab had no influence on multiplication of CD8^+^ T cell (Fig. 2h), indicating that IL-10 is a major factor in the immunosuppressive effect of the PD1^+^ TAMs on CD8^+^ T-cell proliferation.

### Exosomes secreted by tumor induce in vitro expansion of PD1^+^ TAM from monocytes

**Fig. 3:**
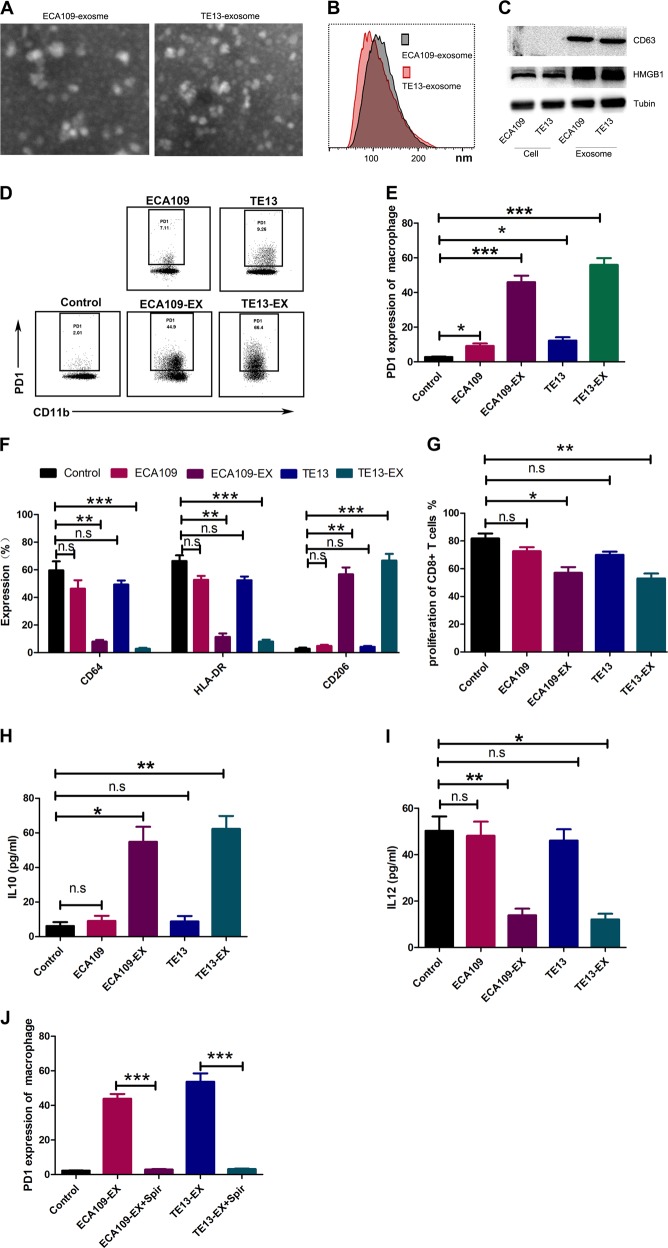
Exosomes derived from tumors induce monocytes into PD1^+^ TAM in vitro. **a**–**c** Exosomes were identified using an electron microscope, Nano-sight, and through western blotting. **d**, **e** On day 3, monocytes grown in presence or absence of supernatant or exosomes were enumerated with the help of flow cytometry to estimate the expression of PD1. Representative graphs are shown in **d**. **e** Data stand for the average of the three experiments performed independently. **f** On day 3, monocytes cultured in presence or absence of supernatant or exosomes were evaluated with the help of flow cytometry for evaluating the frequency of CD64, HLA-DR, and CD206 in monocytes. Data stand for average of the three experiments conducted independently **b**. **g** Estimation of the repressive property of monocytes cultured in presence or absence of supernatant or exosomes with the help of flow cytometry. Results stand for the mean of the three experiments carried out independently. **h**, **i** Estimation of IL-10 (**h**) and IL12p70 (**i**) levels in monocytes cultured in presence or absence of exosomes by ELISA. Data represent the average of three independent experiments. **j** On day 3, monocytes cultured in the medium with exosomes collected from ESCC cells in presence or absence of the exosome release inhibitor spiroepoxide were determined the PD1 level with the help of flow cytometry. *p < 0.05, **p < 0.01, ***p < 0.001 (Student’s *t*-test)

To investigate the effect of ESCC-derived exosomes on PD1^+^ TAM differentiation, we used exosomes isolated from ESCC cell lines to treat monocytes. We confirmed exosome isolation by different techniques such as Nano-sight, western blotting, and flow cytometry wherein we detected presence of the exosomal marker CD63 (Fig. 3a–c). Our results showed that tumor-derived exosomes upregulated the expression of PD1 and CD206 (Fig. 3d–f), and downregulated the expression of CD64 and HLA-DR in monocytes (Fig. 3f). Moreover, monocytes exposed to exosomes showed tendency to suppress proliferation of CD8^+^ T cell (Fig. 3g). Exposure of monocytes to exosomes also enhanced expression of IL-10 (Fig. 3h) and a decrease in IL12p70 expression (Fig. 3i). However, ESCC-derived supernatant could not promote PD1^+^ TAM differentiation (Fig. 3d–i). Importantly, when we treated monocytes and ESCC cells co-cultures with spiroepoxide (exosome release inhibitor), there was a marked decrease in the percentage of PD1^+^macrophages (Fig. 3j).

### HMGB1 is required for exosome-mediated PD1^+^ TAM expansion

**Fig. 4:**
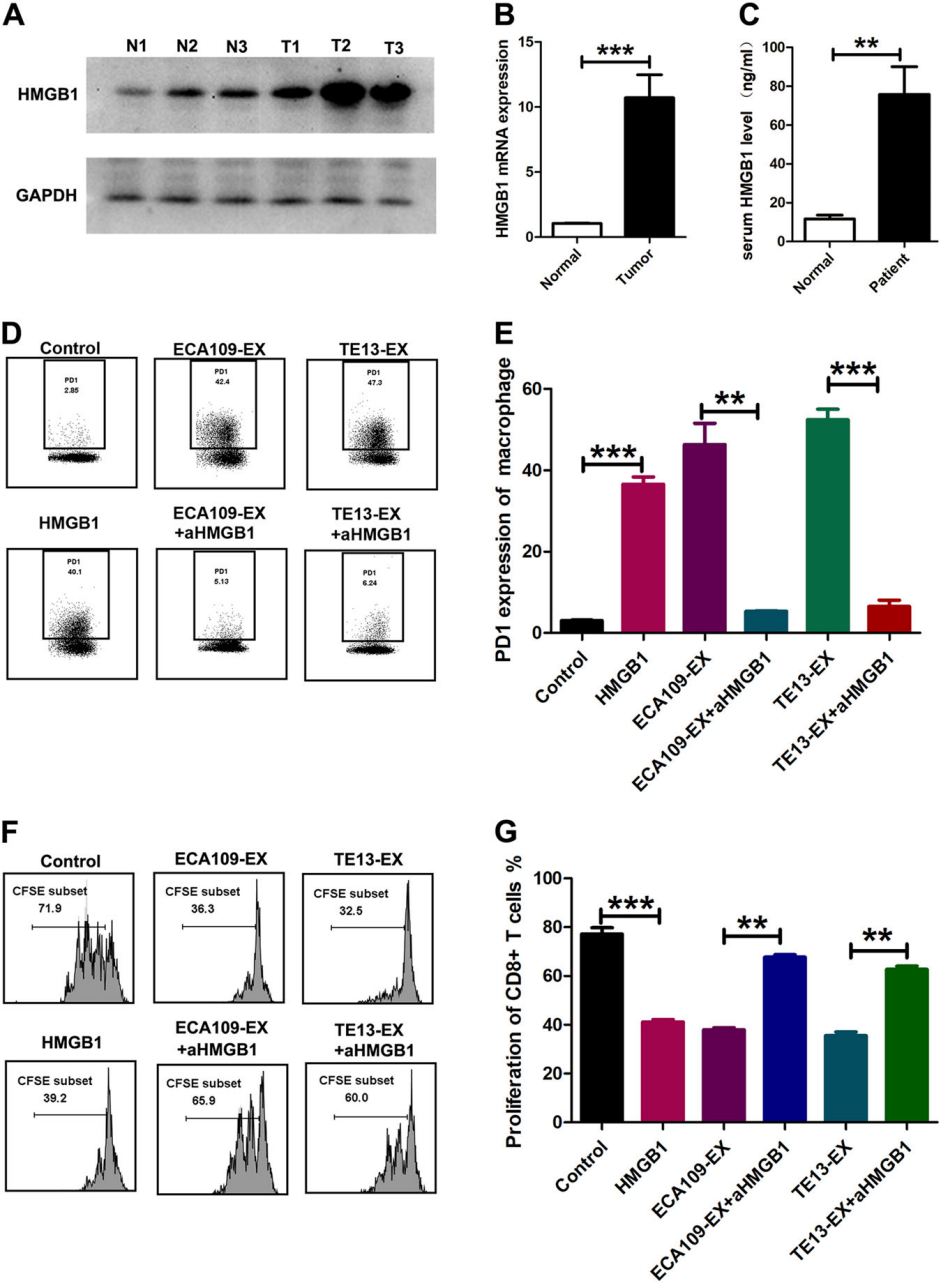

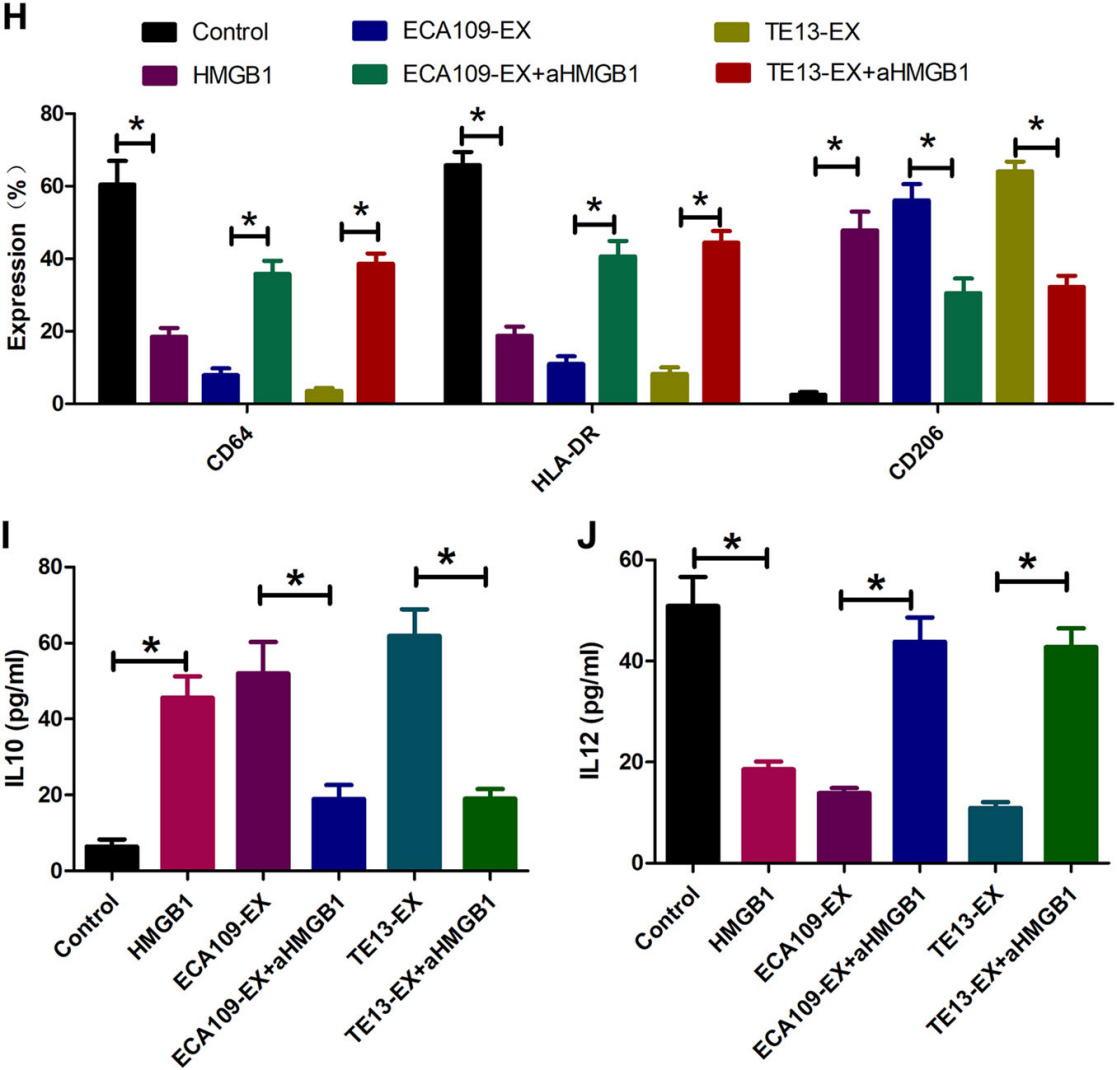
HMGB1 is required for exosome-mediated PD1 ^+^ TAM expansion. **a**, **b** HMGB1 protein expression was measured through western blot analysis (**a**) and qPCR (**b**) in paired ESCC tissues and non-tumor tissues (N, non-tumor tissues; T, tumor). **c** Serum HMGB1 levels were measured using ELISA in a healthy person and in patients with ESCC. **d**, **e** On day 3, monocytes cultured in the medium with or without exosomes or HMGB1 in the presence or absence of aHMGB1 were subjected to flow cytometric analysis to gauge the expression of PD1 in monocytes. Cross-sectional graphs are displayed in **d**. **e** Data stand for the average of three independent experiments. **f**, **g** Estimation of the inhibitory capability in monocytes cultured in the medium with or without exosomes or HMGB1 with or without aHMGB1 by flow cytometry. Representative graphs are shown in **f**. **g** Data mean the average of the three experiments conducted independently. **h** On day 3, monocytes cultured in presence or absence of exosomes or HMGB1 with or without aHMGB1 were evaluated the levels of CD64, HLA-DR, and CD206 in monocytes by utilizing flow cytometry. Data stand for the average of three independent experiments. **i**, **j** Estimation of IL-10 (**i**) and IL12p70 (**j**) level in monocytes cultured in the medium with or without exosomes or HMGB1 by ELISA. Data mean the average of the three experiments carried out independently. *p < 0.05, **p < 0.01, ***p < 0.001 (Student’s *t*-test)

Our next endeavor was to determine the factors involved in the induction of PD1^+^ TAMs by ESCC environments. Recent studies have suggested that HMGB1 released by malignant cells contributes to the differentiation and pro-tumorigenic functions of MDSC in mice (25164013). Indeed, we also observed a marked increase of HMGB1 in plasma from ESCC blood and tissue (Fig. 4a–c). Expression of HMGB1 was much higher in ESCC-derived exosomes than in ESCC cells (Fig. 3c). To study whether HMGB1 is also responsible for the generation of PD1^+^ TAMs in human ESCC, we initially tested the effect of recombinant human HMGB1 on PD1^+^ TAM expansion. Indeed, HMGB1 effectively induced PD1 expression (Fig. 4d, e). Correspondingly, exposure of monocytes to HMGB1 triggered CD206 expression, inhibited HLA-DR and CD64 expression (Fig. 4h), exhibited stronger suppressive function (Fig. 4f, g), upregulated IL-10 expression, and downregulated IL-12 expression (Fig. 4i, j). Importantly, using a specific neutralizing antibody to abolish the effects of HMGB1 in exosomes from ESCC efficiently inhibited PD1^+^ TAM expansion (Fig. 4d–j).

## Discussion

Our work identified an unknown pro-tumorigenic subpopulation of PD1^+^ TAM and applied a multiple analysis approach to study the phenotype, mechanisms of activation, biological function, and clinical significance of the cells of interest in the ESCC microenvironment. Our results confirmed that an unrecognized pro-tumorigenic macrophage subset express PD1 in a constitutive manner, accumulate in advanced stage ESCC, and is negatively associated with the survival of ESCC patients. These PD1^+^ TAMs exhibit a surface profile and function similar to that of M2. In addition, ESCC-derived exosomes promoted PD1^+^ TAM expansion via HMGB1 in vitro.

PD1 is one immune checkpoint drug target that has been clinically extremely successful. The principal function of PD1 involves regulation of T-cell function. However, our previous study, as well as other studies, confirmed that macrophages express high levels of PD1 during tumor progression and in the context of pathogen infection^15–19^, which impair the function of macrophages. Here, we identified PD1^+^ TAMs in the ESCC microenvironment, which exhibited M2 phenotype and function, as well as promoted ESCC progression.

There are reports that state that TAMs polarize towards either inflammatory M1 or M2(pro-tumor) macrophages following the micro environmental cues^38^; TAM populations are distinguished mainly through their surface molecules and function^39^. M1 macrophages in humans express M1 surface markers, like MHCII, CCR7, CD80, and CD86, and pro-inflammatory cytokines, such as IL-12 and TNFα. In contrast, M2 macrophages express specific surface markers, such as CD163, CD206, and CD204, and anti-inflammatory cytokines, such as IL-10^7,38,40^. Our observations confirmed that PD1^+^ TAMs express surface molecule like M2; had a remarkable upregulation in CD206, IL-6, and IL-10 expression; a lucid reduction in HLA-DR, CD64, and IL-12 expression; and exhibited the strong ability to impair CD8^+^ T-cell proliferation. On the contrary, PD1^−^ TAMs showed a drift towards an M1-like surface molecule and function. Importantly, PD1 was not only a surface marker, but also served as a functional marker because triggering this molecule with a functional antibody-enhanced IL-10 expression and the immunosuppressive function of the PD1^+^ TAMs, which is depended on IL-10.

We tried to determine the mechanism underlying the assembly and triggering of PD1^+^ TAMs. The immune-regulatory functions of exosomes are increasingly coming to forefront^24–27^. We hypothesized that tumor-derived exosomes can be pivotal for influencing macrophage polarization during development of ESCC. Indeed, exposure of monocytes to exosomes derived from ESCC caused elevated proportion of PD1^+^ TAMs. Further, the monocytes primed with tumor-derived exosomes showed following distinct features: a relevant increased of CD206 and IL-10; a clear reduction of HLA-DR, CD64, and IL-12, whereas an significant increased capacity to suppress the proliferation of CD8^+^ T cells. However, ESCC-derived supernatant could not promote PD1^+^ TAM differentiation. Importantly, exosome release inhibitor spiroepoxide addition to monocytes and ESCC cells co-cultures caused distinct suppression in the fraction of PD1^+^ macrophages. Thus, ESCC-derived exosomes is a key factor in the production of PD1^+^ macrophages.

HMGB1 serves as an “alarmin” that is often released from stressed and dying cells, also cancer and immune cells^41–44^. Our data confirmed that a marked increase in HMGB1 was observed in plasma from ESCC blood and tissue, and HMGB1 significantly accumulated in tumor-derived exosomes. HMGB1 effectively induced PD1 expression in monocytes. Correspondingly, exposure of monocytes to HMGB1 triggered CD206 expression, inhibited HLA-DR and CD64 expression, exhibited stronger suppressive function, upregulated IL-10 expression, and downregulated IL-12 expression; this suggests that HMGB1 can promote PD1^+^ TAM expansion. Importantly, using a HMGB1-neutralizing antibody to abolish the effects of exosomes from ESCC efficiently inhibited PD1^+^ TAM expansion. These data suggest that ESCC-derived exosomes promote PD1^+^ TAM proliferation via HMGB1 in vitro.

Our work explores into the possible regulation of HMGB1/PD1^+^ TAM-mediated ESCC progression. The work paves way for future therapeutics which target pathological PD1^+^ TAMs and HMGB1 signal for novel strategies for ESCC therapy.

## Materials and methods

### Isolation of human mononuclear cells

Human ESCC samples from resected ESCC were obtained from Lanzhou University Second Hospital. The patients did not undergo anticancer therapy prior to surgery. Individuals with concurrent HIV, other cancer or autoimmune conditions were omitted from our study. Each of the 57 patients gave their consent for our research and the procedure was sanctioned from the Review Board of Lanzhou University Second Hospital. ESCC tissues were cut into small sizes (~0.1 cm) and propagated in Roswell Park Memorial Institute (RPMI) 1640 Medium supplemented with 15% fetal bovine serum (FBS) (HyClone, USA), 0.01% DNase I (Roche, Switzerland) and 0.08% type IV collagenase (Sigma-Aldrich, St. Louis, USA), and for digestion. We separated the cells isolated by digestion using 150-μm filters, while the mononuclear cells (MCs) were isolated through density-gradient centrifugation. Subsequently, MCs were suspended with PBS having 1% FBS (heat-inactivated), and subjected them to flow cytometry analysis. PD1^+^ TAMs were obtained from MCs through flow cytometry and were subjected to functional analysis. The sorting of PD1^+^ TAMs was performed in the basis of CD45, CD11b, F4/80, and PD1 expression.

### Flow cytometry

We procured the following antibodies from BioLegend (San Diego, CA, USA) PE-, BV421-, APC-, or FITC-conjugated mouse anti-human Abs: CD11b (#101212), CD45, PD1 (#329906), CD8 (#344704/344722), CD206 (#321110), HLA-DR (#307606), CD4 (#300508/300514), and CD64 (#305008). We suspended cultured cells or MCs in PBS with 0.1% bovine serum albumin followed by labeling with either particular/isotype Abs for 30 min. at 4 °C Subsequently, 1% paraformaldehyde was used to fix the cells after two PBS washes. We used FACScan and flow jo software for analysis of flow cytometry data.

### Enzyme-linked immunosorbent assay (ELISA)

We analyzed human IL-10 and IL12p70 concentrations of the macrophage-derived culture media using ELISA kits following manufacturer’s instructions (eBioscience for IL12p70 and IL-10).

### Immunofluorescence

5-μm sections were obtained from paraffin-embedded ESCC tissues and subjected to immunofluorescence based on a two-step protocol. We stained the sections rabbit anti-human PD1 (R&D Systems) and mouse anti-human CD68 (Dako) antibodies at 4 °C for duration of 16 h, and then were fluorescently conjugated with secondary Abs for duration of 30 min at 37 °C.

### T-cell proliferation assays

5 × 10^6^ CD8^+^T cells were labeled carboxyfluorescein succinimidyl ester (CFSE; 25 μM, Invitrogen, Carlsbad, CA, USA) at 37 °C for 5 min, followed by two washes, and plated at 1 × 10^6^ cells in a 96-well round plate containing 200 μL of RPMI 1640 medium enriched with 10% FBS. We used fluorescent cell sorting to separate PD1^+^ TAMs from neoplasm tissue and then co-cultured with CD8^+^ T cells at a 1:1 ratio. Subsequently, we used 1 μL of anti-CD8 and 2 μL of anti-CD28 beads for 3 days to activate the CD8^+^ T cells. Finally, we studied CD8^+^ T-cell proliferation by flow cytometry. For certain processes, PD1^+^ TAMs were treated with anti-PD1 Ab (goat human-specific AF1086, R&D Systems) and total goat IgGs (R&D Systems) for 24 h, subjected to two washes before they were co-cultured with CD8^+^ T cells at a 1:1 ratio. In some experiments, 1 μg/mL anti-IL-10 or IL-6 Ab (R&D) was added into the culture system.

### Isolation of exosome and culture of monocytes from ESCC

Human ESCC cell lines including CEA109 and TE13 were obtained from the American Type Culture Collection (Rockville, MD, USA). We collected culture medium with serum (devoid of exosomes) from CEA109 and TE13 on day 3 and culture medium was centrifuged at 1500 rpm for 10 min. We further centrifuged the supernatants for 20 min period at 2500 rpm, before filtering them through a 0.22-µm filter. We isolated exosomes using ExoQuick-TC (System Biosciences) as per recommended instructions. We resuspended exosomes in PBS, and estimated total exosome protein by BCA assay. We incubated monocyte culture system with 50 µg of isolated exosomes for 3 days. In some experiment, monocyte cultures were incubated with 10 ng/mL HMGB1 or 1 μg/mL aHMGB1 (sigma) was used an additive in the culture medium for 3 days.

### Statistical analysis

We calculated the dissimilarities between groups using Bonferroni post-test. Pearson’s correlation analysis helped to test whether the groups correlated with each other. *p* < 0.05 was regarded as statistically significant.
